# Growing Role of 3D In Vitro Cell Cultures in the Study of Cellular and Molecular Mechanisms: Short Focus on Breast Cancer, Endometriosis, Liver and Infectious Diseases

**DOI:** 10.3390/cells13121054

**Published:** 2024-06-18

**Authors:** Nora Bloise, Marialaura Giannaccari, Giuseppe Guagliano, Emanuela Peluso, Elisa Restivo, Silvia Strada, Cristina Volpini, Paola Petrini, Livia Visai

**Affiliations:** 1 Molecular Medicine Department (DMM), Centre for Health Technologies (CHT), Unità di Ricerca (UdR) INSTM, University of Pavia, 27100 Pavia, Italy; marialaura.giannaccari01@universitadipavia.it (M.G.); emanuela.peluso01@universitadipavia.it (E.P.); elisa.restivo01@universitadipavia.it (E.R.); silvia.strada01@universitadipavia.it (S.S.); cristina.volpini01@universitadipavia.it (C.V.); 2 UOR6 Nanotechnology Laboratory, Department of Prevention and Rehabilitation in Occupational Medicine and Specialty Medicine, Istituti Clinici Scientifici Maugeri IRCCS, Via Maugeri 4, 27100 Pavia, Italy; 3 Interuniversity Center for the Promotion of the 3Rs Principles in Teaching and Research (Centro 3R), Operative Unit (OU) of University of Pavia, 27100 Pavia, Italy; 4 Department of Chemistry, Materials, and Chemical Engineering “G. Natta”, Politecnico di Milano, P.zza L. Da Vinci 32, 20133 Milan, Italy; giuseppe.guagliano@polimi.it (G.G.); paola.petrini@polimi.it (P.P.); 5 Interuniversity Center for the Promotion of the 3Rs Principles in Teaching and Research (Centro 3R), Operative Unit (OU) of Politecnico di Milano, 20133 Milan, Italy

**Keywords:** cellular and molecular mechanism, 3D in vitro model, 3D cell cultures, breast cancer, endometriosis, hepatic environment, bacterial infections

## Abstract

Over the past decade, the development of three-dimensional (3D) models has increased exponentially, facilitating the unravelling of fundamental and essential cellular mechanisms by which cells communicate with each other, assemble into tissues and organs and respond to biochemical and biophysical stimuli under both physiological and pathological conditions. This section presents a concise overview of the most recent updates on the significant contribution of different types of 3D cell cultures including spheroids, organoids and organ-on-chip and bio-printed tissues in advancing our understanding of cellular and molecular mechanisms. The case studies presented include the 3D cultures of breast cancer (BC), endometriosis, the liver microenvironment and infections. In BC, the establishment of 3D culture models has permitted the visualization of the role of cancer-associated fibroblasts in the delivery of exosomes, as well as the significance of the physical properties of the extracellular matrix in promoting cell proliferation and invasion. This approach has also become a valuable tool in gaining insight into general and specific mechanisms of drug resistance. Given the considerable heterogeneity of endometriosis, 3D models offer a more accurate representation of the in vivo microenvironment, thereby facilitating the identification and translation of novel targeted therapeutic strategies. The advantages provided by 3D models of the hepatic environment, in conjunction with the high throughput characterizing various platforms, have enabled the elucidation of complex molecular mechanisms underlying various threatening hepatic diseases. A limited number of 3D models for gut and skin infections have been developed. However, a more profound comprehension of the spatial and temporal interactions between microbes, the host and their environment may facilitate the advancement of in vitro, ex vivo and in vivo disease models. Additionally, it may pave the way for the development of novel therapeutic approaches in diverse research fields. The interested reader will also find concluding remarks on the challenges and prospects of using 3D cell cultures for discovering cellular and molecular mechanisms in the research areas covered in this review.

## 1. Introduction

In today’s fast-paced world of life science and biomedical research, a large body of experimental evidence has clearly demonstrated the enormous potential of three-dimensional (3D) cell cultures for improving our understanding of cell biology and the molecular mechanisms underlying disease [1,2,3], for drugs development and testing [4,5,6,7], in regenerative medicine [8,9] and also in tissue engineering [1]. Research has focused heavily on developing protocols and fine-tuning new technological approaches to developing diverse 3D in vitro models, also in agreement with the 3Rs (Replacement, Reduction, Refinement) principles of the European Union [10]. For years, two-dimensional (2D) cell culture systems have been used extensively in biomedical research to study cellular and molecular mechanisms in both physiological and pathological conditions and to develop new therapies and treatments. Despite their low cost and the possibility of high-throughput analysis, 2D cultures do not mimic typical tissue architecture, limiting cell–cell and cell–extracellular matrix (ECM) interactions or the correct oxygen and nutrient gradients that are responsible for the activation of specific cellular and molecular events involved in the underlying biological processes and responsible for the cellular phenotype [10,11]. 3D cell culture platforms can overcome the limits of both 2D cultures and animal models [12,13], providing the conditions for establishing the critical characteristics of the in vivo environment to reproduce the complexity typical of healthy or diseased tissues, thus enabling a better understanding of the underlying cellular and molecular mechanisms [14]. Reviewing the literature makes clear that the enormous and unprecedented potential of 3D cultures is helping to improve the biological relevance of cell lines, which are widely used in scientific research for their stability, reliability and degree of batch-to-batch standardization [15,16,17], by increasing their ability to behave in a manner similar to physiological behavior [18,19]. Similarly, the 3D system allows for the improved phenotypic stability of primary cells, long-term expansion and differentiation into multiple lineages of induced pluripotent stem cells (iPSCs), offering significant advantages over current approaches [20,21]. The ability to obtain a high degree of cell organization, cell–cell interactions and ECM components from all these cell types is closely linked to the use of methods and technologies developed to facilitate the formation of 3D cell models capable of mimicking the complexity of tissues and organs. Spheroids and organoids represent the two most important structures where cells are cultured in 3D [22]. The first is the simplest model of 3D organization, composed of cellular aggregates primarily formed via cell-to cell adhesion derived from cell lines, primary cells or tumor biopsies in mono- or co-cultures. Despite their lower complexity structurally, they have applications in drug and nanoparticles screening and disease modeling, such as for tumors [23]. They can be generated by methods that force the formation of cell aggregation with spherical cells, in the absence or presence of biomimetic and modulable scaffolds/hydrogels (natural or synthetic). The latter are able to provide biomimetic structures capable of recapitulating the physical and biochemical cues required for the cell to adhere and grow to form the desired tissue features [24]. Compared to spheroids, organoids are highly complex self-organized 3D structures derived from the self-organizing properties of stem cells (embryonic stem cells (ESCs), induced pluripotent stem cells (iPSCs), adult stem cells (ASCs) and even tumor cells. These amazing 3D constructs harbor multifarious cell types of original organs and mimic the derived organs in both architecture and function to a great degree [25]. Until now, the generation of organoids from tissue-derived cells (TDCs) or induced pluripotent stem cells (iPSCs) has involved many optimized step-by-step protocols that typically take several months [26]. Patient/tissue-derived organoids or tumor organoids are obtained through optimized tissue dissociation methods (mechanical and enzymatic dissociation can be combined to generate better cell yields) to isolate starting cell populations (tissue–resident stem/progenitor cells or tumor cells), while iPSC-derived organoids are established from fully characterized iPSC lines. The maturation of organoids requires the seeding the cells onto a specific matrix (biologically derived, such as a Matrigel or synthetic hydrogel) and adding specific growth factor cocktails at each step [27,28,29]. Organoids can be also obtained by using spinning bioreactors (rotating vessel bioreactors, clinostat bioreactors and stirred-tank bioreactors), which partially resolve the inadequate nutrient and waste diffusion typically observed in 3D cell cultures [30]. Despite some limitations (e.g., the protocols are not standardized globally; microenvironment components are missing, particularly in organoids derived from adult stem cells; and they are difficult to adapt to microplates and high-throughput screening contexts), organoids are very promising tools for tissue engineering, regenerative medicine, cancer research, new drug screening and personalized therapies. Advances in the development of spheroids and organoids and their application in various fields of research, including clarifying cellular and molecular aspects, have been enabled by the rise of new technological approaches frameable within high-throughput experimental workflows, such as 3D bioprinting or microfluidic techniques [31,32]. 3D bioprinting uses bio-ink, comprising living organoids or spheroids encapsulated within tunable-biomaterial, to precisely create 3D biological geometries mimicking those of the native tissue in a layer-by-layer approach [33]. This technology has rapidly emerged as a promising tool for the creation of 3D cell models with a well-defined architecture, composition and high reproducibility, particularly useful for tumor–stroma investigation and drug screening applications. Microfluidic is another bioengineering approach that rapidly gained attention in cell cultures [34]. It is based on platforms in which living cells are cultured in small micrometer chambers and the medium is continuously infused inside the chambers. With the ability to manipulate flows in the order of a few μL/min, the nutrient supply, the oxygen exchange and the removal of waste products (e.g., cellular debris) can be regulated in a spatially controlled manner. This facilitates both the formation of uniformly sized spheroids (homotypic or heterotypic) and perfused organoids, even dispersed in natural/synthetic scaffolds, as well as the study of growth factors or drug effects and the mechanobiology of cell–cell and cell–matrix interactions, by exploiting the possibility of working in a biomimetic physiological environment [35,36]. Microfluidics have also opened up the development of miniaturized cell models, known as organ-on-a-chip models, which combine different organoids with the ultimate goal of better reflecting the physiology of the human organ [37]. The interested reader may consult the recent reviews in [22,38,39] describing the advantages and limits for obtaining 3D models and also the strategies for overcoming them.

The exciting power of 3D cell cultures is currently being exploited in so many different research fields that it is not possible to provide a complete and comprehensive review. For that reason, the review will emphasize some significant contributions of 3D models in expanding knowledge on the most common cancer in women, breast cancer (BC), and on a non-cancerous but emerging and disabling disease of the female reproductive system, endometriosis. New cellular and molecular aspects obtained by reproducing the complex of the liver in 3D vitro 3D cultures, an organ which plays a central role in metabolic functions, will also be highlighted. Finally, interesting insights that 3D technology has brought to light in the study of bacterial infections will be reported.

## 2. 3D Culture Models for Breast Cancer

**Figure 1:**
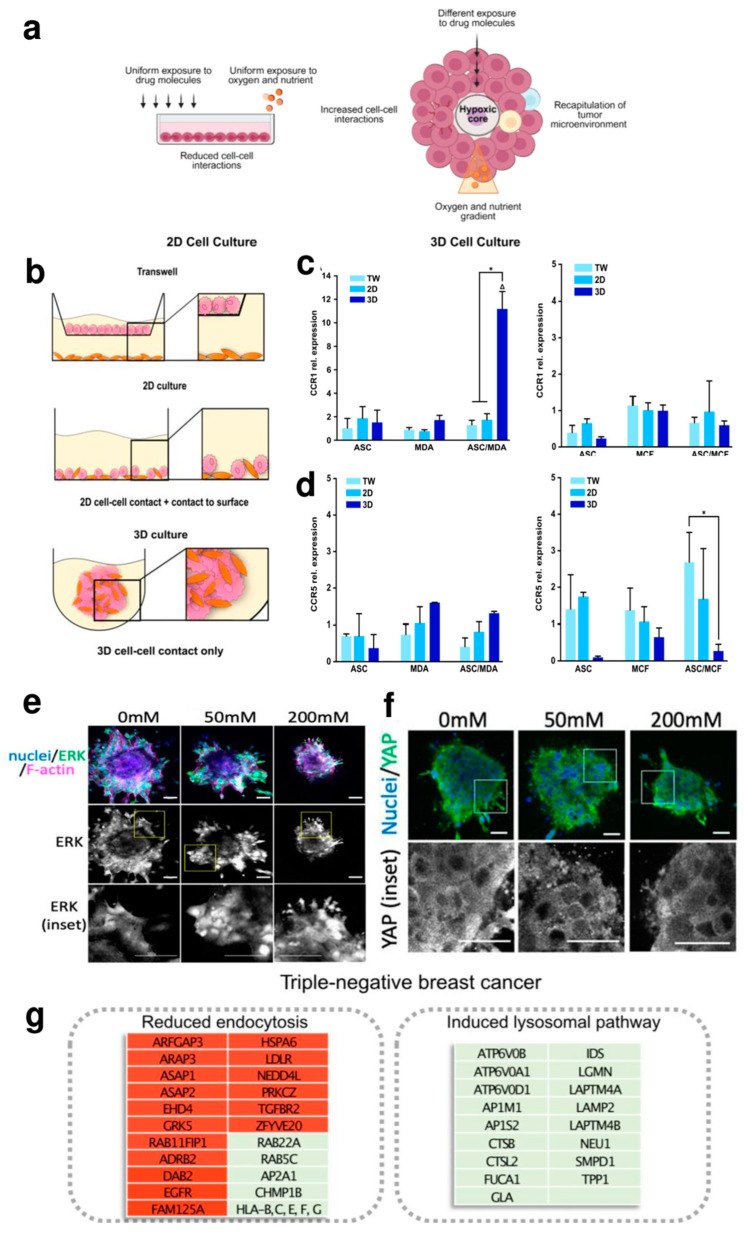
(**a**) In 2D adherent cultures, cells grow as a monolayer on a flat surface, allowing unrestricted access to a similar number of nutrients and growth factors in the culture medium, resulting in homogeneous growth and proliferation. Cell–cell interactions and the extracellular environment are absent. The 3D model recapitulates the characteristics of the tumour microenvironment. Adequate cell–cell and extracellular environment interactions are allowed. A variable availability of oxygen, nutrients, metabolites and signalling molecules is established (adapted from [82] under the terms and conditions of the Creative Commons Attribution (CC-BY) license (CC-BY 4.0)). (**b**–**d**) Schematic representation of different culture conditions (**b**), expression of CCL5 receptors (CCR1 and CCR5) in mono- and co-culture spheroids of ASCs and MDA-MB-231 or MCF-7 compared to indirect and direct 2D cultures (**c**,**d**). * indicates statistically significant differences (*p* < 0.05) between culture systems; Δ indicates statistically significant differences (*p* < 0.05) to corresponding monocultures (adapted from [56] under the terms of the CC-BY 4.0 publishing license). (**e**,**f**) Images of HCC1954 spheroids stiffened by different concentrations of ribose (0.50 and 200 mM). (**e**) Fixed samples show the distribution of ERK (green) and F-actin (magenta), with counterstained nuclei in blue. Scale bars, 20 μm. (**f**) Spheroids embedded in a 3D collagen structure show the localization of YAP (green); nuclei are stained blue. Scale bars, 20 μm (adapted from [57] under the terms of the CC-BY 4.0 publishing license). (**g**) Box representation of doxorubicin effects in the MDA-MB-231 cell line cultured within the 3D biomimetic collagen scaffold, indicating the most significantly altered pathways implicated in DOX resistance (green = up-regulation; red = down-regulation) (adapted from [64] under the terms of the CC-BY 4.0 publishing license).

In the modern industrialized world, cancer has become the most feared disease, representing one of the main causes of death after cardiovascular diseases [40]. Currently, there is an ever-growing plethora of scientific articles reporting on the use of 3D cultures in oncological research of both so-called ‘solid’ [14,41] and ‘liquid’ tumors [42,43]. The shift from 2D in vitro systems to 3D cultures is explained by the fact that it is possible to tune these to more closely mimic in vivo tumor characteristics, including the heterogeneity of the tumor microenvironment (TME) [44], cell–cell (i.e., tumor cell–immune cell) [45] and cell–extracellular matrix contacts [46], hypoxia [47], nutrient and pH gradients [48] and biomechanical properties (such as extracellular matrix (ECM) stiffness) [49] (Figure 1a). This cancer-mimicking approach facilitates the study of mechanisms of cancer initiation, progression, resistance recurrence and tumour–stroma interaction, which can identify markers for early diagnosis and therapies [41,50]. At the same time, 3D cultures provide a tool for predicting therapeutic responses, optimizing treatment strategies and exploring potential therapies in the battle against cancer [23]. 

In this section, we briefly focus on some recent findings using 3D cell cultures to understand the mechanisms underlying BC, the most common cancer in the world [51]. BC is a heterogeneous disease determined by both genetic and environmental factors and is categorized into different subtypes based on the levels of the receptors for estrogen, progesterone and human epidermal growth factor 2 (HER2) and the absence of the aforementioned receptors [52]. Since the inter- and intra-heterogeneity of breast tumours complicates their treatment, it is very relevant to have systems in place for identifying, clarifying, and defining changes in cellular properties for each type and stage of BC. Currently, xenografts and syngeneic in vivo breast cancer models are most used to test the in vivo efficacy of new treatments before entering clinical trials., but each has advantages and disadvantages [53]. As recently reviewed by Fröhlich et al. [39], to increase translation from in vitro findings to a clinical setting, many 3D models are available, including, e.g., spheroids, organoids and breast cancer on-a-chip and bio-printed tissues. The literature on the subject is vast. In this review, we have mainly focused on some recent articles that, by culturing immortalized breast cancer cell lines and other key TME cells, such as cancer-associated fibroblasts (CAFs) and adipocytes (ASCs), on 3D platforms, have contributed to shedding light on aspects related to the processes of BC proliferation, migration, invasion, tumour–stroma interaction and drug resistance (Table 1). 

The great potential of 3D models (like spheroids and organoids) and linked methods/technologies is to recreate important features of the tumor, such as the organization of the multicellular layer and the environment in which micro-metastases develop, as nutrients and oxygen are limited in these large structures [24,47]. In addition, they make it possible to represent and preserve the cellular heterogeneity present in tumors: combining different cell types in the same spheroid, i.e., tumor cells, monocytes and CAFs, allows for studying the role of these cells and the cell–cell interaction in tumour initiation and progression as well as all the variations in the signalling, gene expression and protein production pathways involved [14]. CAFs are among the central components of TME, and they promote tumor progression and metastasis [68]. Increasing evidence points to exosomes, small membranous vesicles containing lipids, proteins and nucleic acid (i.e., DNA, mRNA and non-coding RNA like microRNA), as critical cellular communicators involved in the interaction mechanism between CAF and cancer cells [2]. Using spheroid models, Cheng et al. [55] aimed to study in BC the role of miR-500a-5p, whose expression is known to be up-regulated in other cancer types [69]. In this work, the 3D cell culture allowed them to observe that CAFs promote breast cancer progression and metastasis through exosomal miR-500a-5p, which led the authors to hypothesize that CAF-derived miR-500a-5p inhibition could be an alternative modality for treating BC [55]. However, spheroid-based 3D models are often oversimplified and do not reproduce the correct dynamic tumour–stroma interactions at cellular and molecular levels. Microfluidic models in combination with hydrogel-based 3D matrices may help to establish the proper tumour–stroma architecture. Glycoprotein nonmetastatic melanoma protein B (GPNMB) is a transmembrane glycoprotein found to be highly expressed in many types of cancer, including breast cancer, with various roles in tumour invasion, angiogenesis, cell adhesion and immunosuppression [70]. Truong et al. [67] engineered a 3D organotypic microfluidic coculture system of tumor–stroma interactions. RNA-seq profiled the transcriptome of cancer cells in this 3D cell culture, which led to the identification, for the first time in breast carcinoma cells, of the involvement of GPNM in the invasion process promoted by CAFs by exploiting the 3D tumor microenvironment in which interactions with CAFs were present. Dysfunctional adipocyte metabolism also plays a very important role in BC progression, as ASCs interact with the mammary epithelium by secreting a variety of cytokines and hormones [71]. CCR1 is the C-C motif chemokine receptor ligand 5 (CCL5) and has been reported to be a chemokine that fosters metastasis and is expressed during crosstalk between breast cancer and stromal cells [72]. In 2023, Watzling et al. [56], utilizing an ASCs/BC spheroid model, observed a predominant chemokine/receptor interaction between CCL5-producing ASCs and its cognate receptor (CCR1) expressing BC cells in promoting the migration of triple-negative MDA-MB-231 breast cancer cells, proving the crucial role of 3D cell cultures in deciphering BC cells (Figure 1b,c). Regarding BC progression, in 2023, Itah el al., culturing human mammary epithelial MCF-10A.B2 cells in a 3D cell culture, established a tumour suppression function for the JNK signalling pathway in HER2+ BC [65].

The ECM of the BC has a subtype-specific composition that may also contribute to changes in the biophysical properties, such as stiffness, of the BC tissue [53,73,74,75]. Enhanced matrix deposition and realignment of collagen fibres are detected by tumor cells, triggering the epithelial–mesenchymal transition (EMT), which in turn stimulates cell motility and invasiveness. In contrast to conventional 2D cell cultures, in which the cell morphology is constrained in a ‘flat’ plain, spheroids can be surrounded by a matrix that can be shaped and stiffened. In this way, it is possible to study the role of these signals in directing the polarity of tumor cells and, thus, their ability to migrate and invade [76]. Very recently, a group of researchers embedded spheroids of HCC1954 human BC cells in 3D collagen scaffolds with increasing stiffness provided by different concentrations of ribose (0, 50 and 200 mM). They showed that the invasion of BC spheroids is driven by the activity of ERKs (extracellular signal-regulated kinases) and the transcriptional regulator YAP (Yes-associated protein). They observed that ERK activity is increased under high stiffness conditions: it was evident how ECM stiffness improves ERK nuclear localization. (Figure 1e). Similarly, YAP activity is affected by matrix stiffness: like ERK, at high ribose concentrations, spheroids showed more YAP activation and nuclear distribution compared to the citoplasmic compartment (Figure 1f). There is evidence that both YAP and ERK activity may play a key role in ECM remodeling to provide a favorable matrix for BC cell migration [57]. Although the underlying mechanism remains elusive, it is known that the stiffness of the ECM matrix influences cell behavior through cancer metabolism regulation [77,78]. Interestingly, applying a well-defined hybrid hydrogel system mimicking the heterogeneous local stiffness of TME, Liu et al. showed that BC cells proliferate in a soft core environment while migrating in a stiff peripheral environment. Furthermore, they observed that BC cells shift from glycolysis to OXPHOS and fatty acid metabolism, responding to a stiff matrix microenvironment [66].

3D cell cultures helped to investigate the BC lineage-specific resistance mechanisms [60,61,62,64]. In these systems, tumor cells display typical tumor characteristics (relative quiescent state, self-renewal capacity and growth in spheroid structures), live in hypoxic and nutrient-poor conditions and express specific ECM components [79]. All these aspects might influence the response to chemotherapy by limiting drug accessibility in the cancer cells [80]. In this way, the evaluation of drug resistance in 3D cell culture models can be valuable in gaining insight into the general and specific mechanisms of drug resistance, while providing more physiologically relevant systems for disease modelling and drug screening. Doxorubicin is considered a first-line anticancer drug in several types of cancer, but drug-induced cardiotoxicity and drug resistance are the main obstacles to its use [81]. In 2022, using a 3D model, Liverani et al. [64] attempted to explain the mechanisms behind these barriers. Specifically, they engineered a 3D model based on biomimetic collagen scaffolds, revealed the involvement of hypoxia in doxorubicin resistance in MDA-MB-231 and also made it possible to identify the most significantly altered pathways involved in drug resistance (Figure 1g). As mentioned above, there are numerous examples of the great potential of 3D models in the study of breast cancer. 

The articles cited in this brief section help to demonstrate how 3D models can be effectively used to understand tumour cell–TME interactions and their impact on drug sensitivity through the ability to mimic cancer characteristics.

## 3. 3D Culture Models for Endometriosis

**Figure 2:**
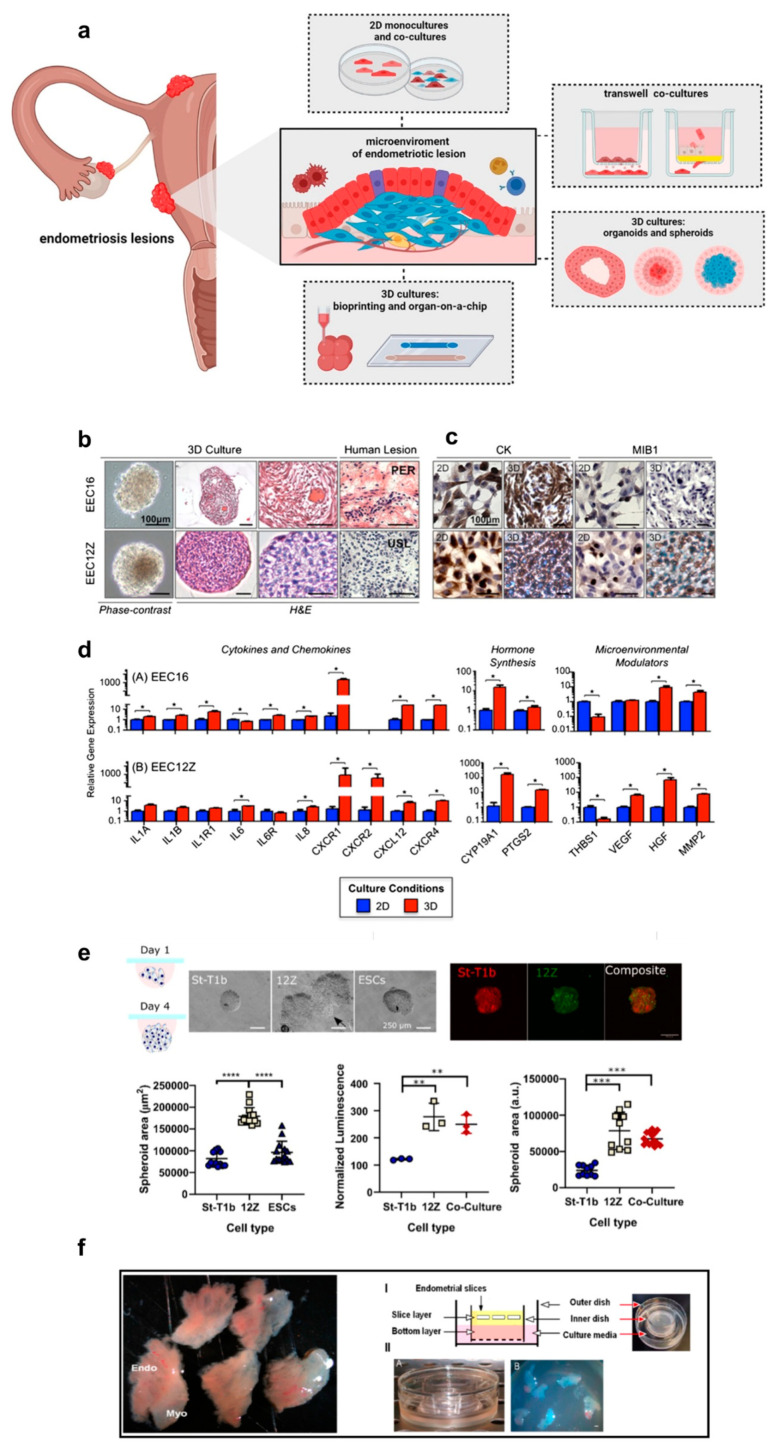
3D culture models of the human endometrium. (**a**) Available in vitro experimental systems used in endometriotic studies, which reflect the multifactorial nature of the endometriotic lesion (adapted from [83] under the terms of a CC-BY 4.0 publishing license). (**b**,**c**) After 7 days of the 3D culture, EEC12Z and EEC16 both form dense, smooth and symmetrical spheroids. (**b**) Phase contrast and H&E images, (**c**) Cytokeratin expression is increased in 3D models versus that in 2D models and (**d**) Expression of genes relevant in endometriosis in EEC16 and EEC12Z after being cultures in 3D for 7 days. * *p* > 0.05. (adapted from [86] under the terms of a CC-BY 4.0 publishing license). (**e**) Spheroids’ shape and dimension characterization: bright-field images (Scale bars 250 µm), Cell Tracker staining (Red: St-T1b; green: 12Z. Scale bar 200 µm) and relative quantitative analysis. ** *p* < 0.01; *** *p* < 0.001, **** *p* < 0.0001; (adapted from ref. [87] under the terms of a CC-BY 4.0 publishing license). (**f**) Representative images showing a 3D cell culture model system for endometriosis based on a slice from a full-thickness human endometrium (adapted from ref. [90] under the terms of a CC-BY 4.0 publishing license).

A large body of current literature showed that the 3D microenvironment can be exploited to better understand cell–cell interactions in endometriosis [83]. Endometriosis is an inflammatory gynaecological disease that seriously affects the quality of a woman’s life. The pathogenesis of endometriosis is still unknown, but several leading theories include retrograde menstruation, altered immunity, coelomic metaplasia and metastatic cell spread [84]. The heterogeneity and differences among the three main classes of endometriosis presentation may suggest different multiple pathogenetic pathways [85]. Over the years, the vitro 3D culture models of the human endometrium have been gradually developed (Figure 2) as an alternative to classical 2D culture models [83]. Currently, several cell types are used in these models, including epithelial, stromal, endothelial and immune cells, as described in Table 2. 

Both the immortalized cell lines and primary cells from healthy women or women with endometriosis were tested to construct the 3D model. In the endometriosis 3D model, organoids, chicken chorioallantoic membranes (cams), amniotic membranes, spheroids and organs-on-a-chip were used (Figure 2a) [83]. Due to the heterogeneity of pathogenesis, the main molecular mechanisms examined by 3D models concern the immunological aspects [86,89], the hormonal signaling [86,90] and the angiogenesis process related to endometriosis [91] and the endometrial stromal cells’ migration and invasion [87]. The models shown in Table 2 demonstrate how genes involved in the above mechanisms are over-expressed in the 3D model compared to those in the 2D model. More in detail, a 3D model based on spheroids has been established to mimic endometriosis using the endometriotic cell line, EEC16 and 12Z [86]. In this example, molecules related to the immune response (including IL6, IL8, CXCL12 and CXCR4), micro-environmental interactions (such as MMP2 and hepatocyte growth factor (HGF)) and hormonal signaling (including prostaglandin-endoperoxide synthase 2 (PTGS2) and cytochrome P450 family 19 subfamily A member 1 (CYP19A1)) were significantly up-regulated compared with the 2D models (Figure 2b–d) [86]. Furthermore, Stejskalová et al. observed the ability of the immortalized eutopic stromal cell line St-T1b to form spheroids (Figure 2e) [87]. Compared with 2D models, the obtained spheroids showed an increased expression of two matrix metalloproteinases (MMPs), MMP2 and MMP14, known to be highly expressed in the early stage of endometriosis [93]. Exploiting 3D cell cultures, the same authors also observed an altered expression pattern of microRNAs miR-200b and miR-145, previously shown to be dysregulated in endometriosis [94], suggesting their involvement in the invasive behavior of the endometriotic epithelial cell line 12Z. Wendel et al. proposed spheroids using 12Z cells to represent the inflammatory (IL6, IL8 and MCP1) and estrogen-related gene (CYP19A1, HSD17β1 and ESR1) expression of endometriosis [88]. These genes were up-regulated in cells grown as spheroids compared to monolayer cultures. Seeking the expression of the same markers described above involved in inflammation (Tumor necrosis factor-TNF), immune responses (interleukins-ILs) and invasion (MMPs), Song et al. created spheroids with endometriotic epithelial and stromal cell lines [89]. Moreover, Muruganandan et al. developed a 3D culture system with an endometrial tissue slice cultured by incorporating an air–liquid interface into a 3D matrix scaffold of type I collagen gel (Figure 2f) [90]. This long-term slice culture method provides a unique in vivo-like microenvironment for studies of human endometrial repair, regeneration and remodeling.

Another example described a microengineered vascularized endometrium-on-a-chip (MVEOC) [91]. The model reconstitutes an endometrial environment including three distinct layers of the epithelium, stroma and blood vessels, and it has demonstrated its appropriate responsiveness to pro-angiogenic factors and hormonal stimulation. Finally, the last example proposed in Table 2 demonstrated that endometriosis also maintains the epigenetic changes in vitro, exploiting organoids as 3D in vitro platforms compared to endometriosis tissue biopsy specimens [92]. These are the most recent examples in the literature describing the applicable 3D model for endometriosis. The ongoing 3D in vitro development will lead to models that accurately reflect the disease phenotype and heterogeneity of this condition. Since endometriosis 3D models more closely resemble the in vivo microenvironment, the potential for identifying and translating novel targeted therapeutic strategies will be greatly enhanced by using these models. 

## 4. 3D Culture Models for the Liver

**Figure 3:**
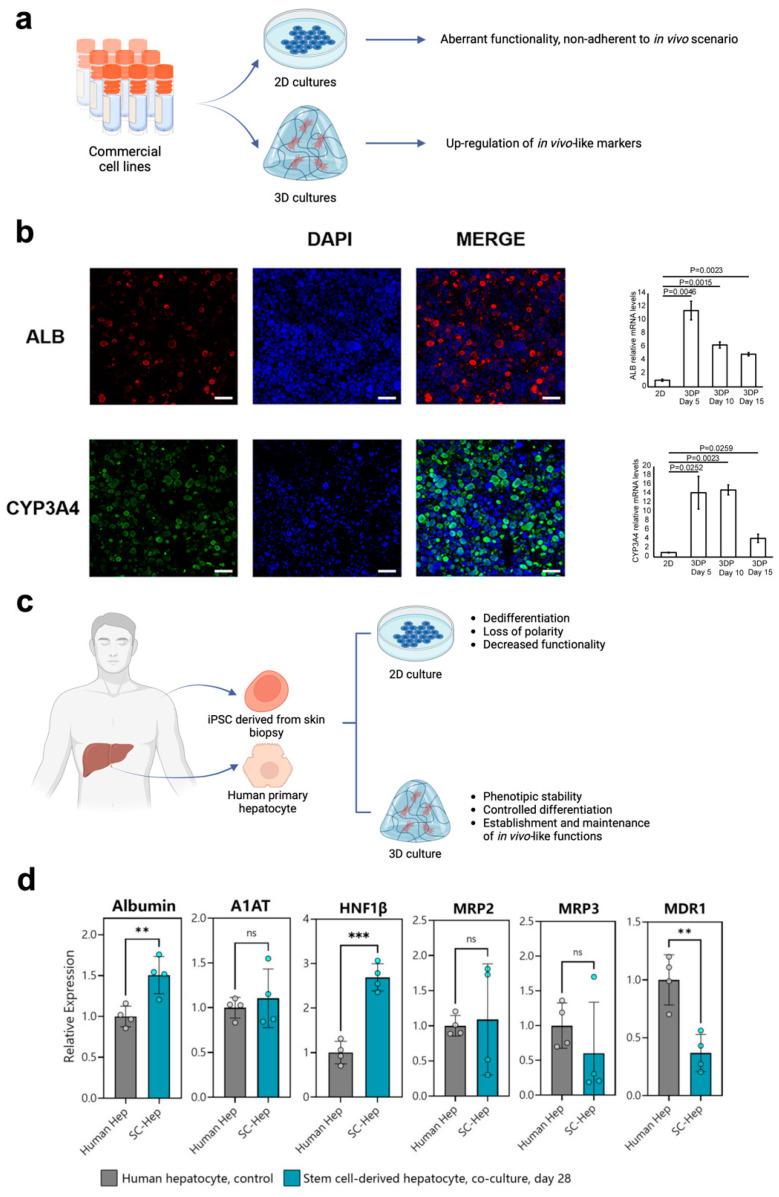
Beneficial impact of growing hepatic cells within a three-dimensional environment. (**a**) When cell lines are cultured in a 3D environment, the systematic up-regulation of in vivo-like functions has been observed. Scheme created with BioRender.com. (**b**) The increase in albumin secretion, as well as the up-regulation of relevant cytochromes, were observed for HepG2 cells grown within a 3D-bioprinted hydrogel matrix, the scale bars are all equal to 200 μm (adapted from [101] under the terms of a CC-BY 4.0 publishing license). (**c**) 3D culture environments positively impact both the establishment and the maintenance of physiological-like hepatic functions in primary hepatocytes, as well as in iPSC-derived hepatocytes. Scheme created with BioRender.com. (**d**) 3D cultures have also shown their potential in guiding the differentiation of iPSC-derived hepatocytes, as the coculture of the iPSC spheroid and primary hepatocytes spheroid led to the establishment of multiple physiological-like hepatic functions. Statistical significance is expressed as ns: *p* > 0.05, **: 0.01 ≤ *p* < 0.001, ***: *p* ≤ 0.001 (adapted from [115] under the terms of a CC-BY 4.0 publishing license).

A notable example of the transition from 2D to 3D models is 3D cultures that mimic the liver environment using both cell lines and iPSCs (Figure 3a,c). Regarding cell lines, HepG2 cells are particularly used. This hepatocyte-like cell line is derived from human hepatocellular carcinoma [15,95]. Different from physiological primary hepatocytes, HepG2 is characterized by an almost null metabolism [96]. This aspect greatly limited its application when modeling the hepatic activity with traditional monolayer cell cultures. However, it was first noticed that when grown within tridimensional environments, the production of albumin and urea was significantly enhanced [97]. The expression of these molecules is a key marker of hepatic metabolism. Usually, HepG2 is characterized by extremely low secretion rates for both molecules [98]. The awareness that their secretion can be boosted by adopting a 3D culture ignited a spark and trailblazed the development of subsequent 3D systems, aiming to understand if the metabolic activity of HepG2 could have been further pushed toward a physiological-like situation. Further studies exploited both scaffold-based cultures and spheroids to investigate to what extent the tridimensionality was responsible for increasing the functionality of HepG2 cells. Particularly, this approach allowed for understanding both qualitatively (immunofluorescence) and quantitatively (RT-qPCR) the relevance of a 3D environment in boosting the expression of relevant metabolic markers such as the secretion of albumin (ALB) and enzymes related to the processing of drugs, such as cytochromes from the cytochrome-P450 family (e.g., CYP 3A4) (Figure 3b) [99,100,101,102,103]. HepG2 cells usually lack the expression of these proteins, and such a behavior might open the door to produce predictive models based on this cell line. Similar findings, resulting from cells being grown in a 3D environment, were also observed for other hepatocyte-like lines, including HepaRG and HUH7 [104,105,106]. Additionally, the strategies currently adopted for fabricating 3D cultures enabled the production of multicellular cocultures. These tools allowed for exploring the impact providing cultured cells with relevant cell–cell interactions and underlined a further boost of the metabolic activity of hepatocyte-like cells when co-cultured with other non-parenchymal cell types (e.g., fibroblasts, stellate cells, endothelial cells) [107,108,109,110,111]. 3D cell cultures emerged as a powerful tool for enhancing the phenotypic stability of both primary hepatocytes and iPSC-derived hepatocytes (Figure 3c). This achievement was possible by providing a culture environment capable of mimicking the native tissue, thus overcoming the lack of compliance that characterizes polystyrene substrates used for monolayer cultures [112,113,114]. Additionally, the possibility of taking advantage of 3D environments to recapitulate cell–matrix interactions was shown to be pivotal for inducing the differentiation of iPSC and other multi-/pluripotent cells into hepatocytes [115,116,117] (Figure 3d). These findings and other relevant contributions (Table 3) are shedding new light on the possibility of successfully growing primary cells in vitro, thus opening pathways that might culminate in highly relevant personalized medicine. 

The advantages provided by 3D models of the hepatic environment, together with the high throughput characterizing these platforms, allowed for elucidating the complex molecular mechanisms behind different threatening hepatic diseases. Particularly, it was recently possible to unveil new aspects featuring the development and progression of hepatocellular carcinoma, including the interactions between the ubiquitin E3 ligase CHIP and transferrin receptor, causing the inhibition of ferroptosis and culminating in enhanced cell proliferation [118]. Similarly, the implications of SIRT7 in promoting the hippo/YAP pathways, thus promoting cancer progression, were recently discovered by exploiting 3D cell cultures [119]. Coherently, 3D in vitro models are playing a crucial role in the identification of novel therapeutic targets. To this end, recent research highlighted that inhibiting macroscopic DNA damages repair by targeting AP-2α with LEI110 leads to the eradication of hepatocellular carcinoma [120]. Research in this sense was not only focused on neoplastic disorders but also on other concerning diseases like hepatic fibrosis. In this sense, it was shown that by promoting PPARγ expression, it was possible to deactivate hepatic stellate cells (which are primarily involved in the production of fibrotic tissue) by inhibiting EZH2-mediated histone H3K27 trimethylation [121].

## 5. 3D Culture Models for Bacterial Infections

Bacterial infections are a leading cause of death worldwide, primarily due to the emergence of antibiotic-resistant bacteria. This poses a significant threat to public health. According to the World Health Organization, deaths caused by antibiotic-resistant strains are projected to surpass those caused by cancer by 2050. Therefore, scientists are endeavoring to enhance their comprehension of infection mechanisms, as host–microbe interactions significantly impact human physiology [122]. At present, many 2D models used to study bacterial infections are deficient due to several factors, including a failure to accurately mimic the in vivo bacterial environment, the lack of pathogen-specific cell types and receptors [123], the absence of cross-talking networks due to the human tissue structure [124] and other factors [125]. Recent discoveries have shown a growing interest in 3D culture models due to their ability to provide more accurate biochemical and biomechanical microenvironments [126]. The development of 3D organotypic models, such as organ-on-a-chip systems, provides a promising platform for modeling physiological and pathological functions of tissues and organs in vitro. These models have been applied in various fields, including the investigation of in vitro bacterial and viral infections. Various 3D models used in the study of bacterial infections are presented in Table 4.

Cheng et al. [127], in their study, bioprinted a gelatin methacryloyl channel with Caco-2 to explore the inflammatory pathways triggered by co-cultures with *Salmonella enterica* and *Lactobacillus reuteri* in aerobic and anaerobic environments. The 3D sacrificially printed gut model has been able to capture the key interactions between the host and the microbes, identifying the enrichments of pathways associated with the inflammatory response. In a separate study, *Gardnerella*, *Prevotella*, *Atopobium vaginae* and *Sneathia amnii* were co-cultured with a representative health-associated commensal, *Lactobacillus crispatus,* using a 3D cell model of the cervix, demonstrating that the four pathogens caused the synthesis of numerous pro-inflammatory chemicals [128]. Furthermore, *S. amnii* strains exhibit potential oncogenic mechanisms based on the altered immunemetabolic microenvironment. Calatayud et al. [129] employed the compartmentalization properties of transwell chambers, reproducing a 3D model of the small intestine and exposing it to synthetic microbiota composed of eight Gram-positive and -negative commensal bacterial strains. The authors studied the different responses in the model due to either the presence or absence of lipopolysaccharide (LPS), a component of Gram-negative bacterial cell walls. They observed that the presence of LPS caused a decrease in epithelial barrier function and an increase in the production of IL-6. Calatayud et al. discovered an increase in the mRNA expression of dual oxidase 2 (DUOX2) and toll-like receptors 2 and 4 (TLR-2, TLR-4) in eukaryotic cells when exposed to LPS and microbiota. DUOX2 is a protein belonging to the NADPH oxidase family and is involved in regulating reactive oxygen species (ROS) in eukaryotic cells, whereas TLRs are membrane receptors involved in host immune defense. The described approach can be utilized as a baseline for subsequent applications, such as the utilization of primary cells or organoids in a coculture with synthetic or natural complex microbial communities, to enhance host–microbiome in vitro research. Recently, organoids have emerged as a promising model for replicating 3D environments in vitro. Gut organoids are currently being used to study bacterial pathogenicity, including *Listeria monocytogenes* [130], which can cross the epithelial barrier and induce an inflammatory response in the epithelium.

**Figure 4:**
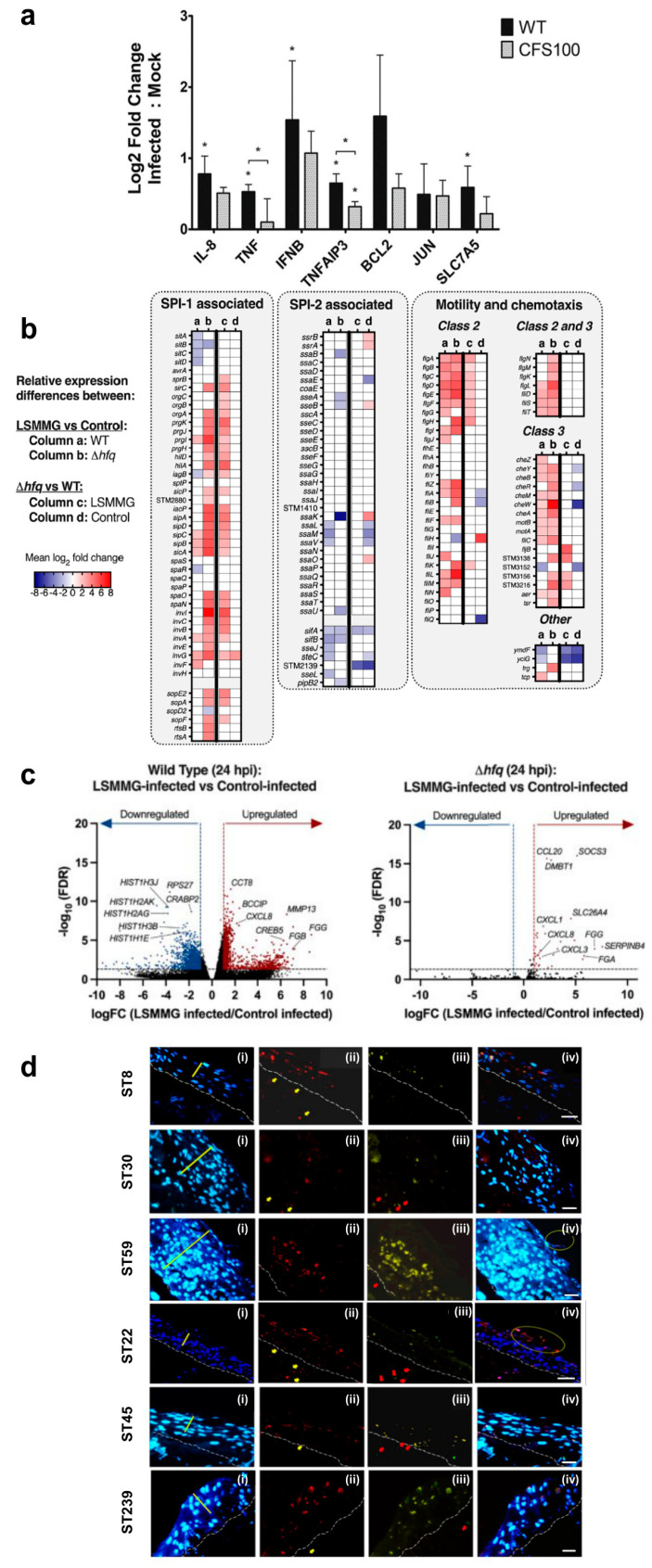
Infection in 3D culture models. (**a**) Human intestinal enteroids (HIE) monolayers were infected with wild-type (WT) Shigella and the avirulent CSF100 strain. The panel shows host gene expression (as log_2_-fold change) after three hours of Shigella infection (adapted from [131] with permission from Copyright American Society for Microbiology-License number 1494825-1). Statistical significance is expressed as *p* < 0.05, *. (**b**,**c**) Salmonella infection in LSMMG and in control culture conditions (adapted from [132] under the terms of a CC-BY 4.0 publishing license). The analyzed strains were the wild-type (WT) and the mutant delta-hfq strains. (**b**) Bacterial genes that were up- and down-regulated, in red and blue, respectively, were associated with Salmonella Pathogenic Islands (SPI) 1, 2, motility and chemotaxis. The expression was reported as the mean log_2_ fold change. (**c**) Plots illustrating the up- (red dots) and down-regulation (blue dots) of genes expressed by host cells infected at 24 h post-infection (hpi) by WT (left panel) and delta-hfq (right panel) strains in LSMMG conditions. Expression reported as the logFC (logged fold change) as a function of the FDR (false discovery rate) < 0.05. (**d**) Label of a section of a skin 3D model after 48 h of infection with MRSA bacterial strains (ST8, ST30, ST59, ST22, ST45, ST239). Each line composed of i, ii, iii and iv represents a bacterial strain. The white dashed line marks the dermal epidermal barrier between the stratum basale and the collagen gel containing fibroblasts. Specifically, (i) shows HaCaT keratinocytes nuclei, at the strata basale and spinosum (yellow line), marked in blue with Hoechst stain. (ii) shows MRSA bacteria labeled with an anti-*S. aureus* antibody and Alexa Fluor^®^ 568 conjugated secondary antibody. Those indicated by yellow arrows are in the collagen gel. (iii) shows the Click-iT^®^ TUNEL Alexa Fluor^®^ 488 cell for the detection of damaged DNA. Finally, (iv) is an overlay where bacteria and apoptosis/DNA damage are co-localized in keratinocytes in the stratum spinosum. The yellow circles in (iv) depict the model’s skin being exfoliated. Scale Bar (i–iv) of 50 µm (adapted from [133] under the terms of a CC-BY 4.0 publishing license).

A study conducted by Koestler et al. [131] reported human intestinal enteroids (HIE) monolayers infected with the virulent *Shigella flexneri* and an avirulent strain (CSF100). In the work, the expression (as fold change) of human host genes involved in inflammation, apoptosis and autophagy was analyzed (Figure 4a). The authors discovered, after the three hours of the infection of HIE with the wild-type (WT) *Shigella*, that, compared to the avirulent CFS100 control strain, there was a significant increase in host inflammation gene expression such as: nuclear factor kappaB (NF-κB), IL-8, interferon beta (INF-β), tumor necrosis factor alpha (TNF-α) and tumor necrosis factor alpha-induced protein 3 (TNFAIP3). The latter gene was significantly increased after infection with the avirulent Shigella, indicating that the control can induce changes in the host response. Furthermore, B-cell lymphoma 2 (BCL-2) and solute carrier family 7 member 5 (SLC7A5) genes, involved in apoptosis and autophagy, respectively, have been discovered to be up-regulated after infection with the wild type of a virulent strain (Figure 4a). HIEs can be powerful 3D models for future investigations of previously unknown features of Shigella pathogenesis, allowing researchers to explore bacterial interactions with the mucin, host immune cells and innate immune responses. A recent study on 3D models of the intestine simulated the initial phase of *Salmonella enterica* (serovar Typhimurium) infection using a low fluid shear culture system designed to mimic microgravity conditions (LSMMG—Low Shear Modeled Microgravity) [132]. In this study, wild-type and delta-hfq mutant Salmonella were cultured in LSMMG and in a control culture in order to investigate the role of the Hfq, an RNA-binding chaperone, in regulating the transcriptional stress response of microorganisms to the LSMMG culture. The authors, Barrila et al. [132], showed that a lack of gravity in LSMMG significantly up-regulated Salmonella virulence proteins involved in host adhesion and invasion. Although the mutant delta-hfq strains were defective in invasion genes, they were expressed in LSMMG. Panel B of Figure 4 reports the different gene expressions (as mean log2 fold change) of the WT and delta-hfq Salmonella strains in LSMMG culture conditions compared to control conditions. In particular, the analyzed genes were associated with the Salmonella Pathogenicity Island (SPI)—1 and 2, motility and chemotaxis [132]. Genes belonging to SPI-1 that are responsible for host colonization, such as invA, invG, prgI, sipC and other genes involved either in motility (flgA, flgB, flgC) or in chemotaxis (cheB, cheM), were up-regulated in WT cultured in LSMMG. These genes were divided into classes 2 and 3 according to either the middle or late assembly stages. Other genes included in SPI-2 such as ssaL, ssaM, sifA steC and the Salmonella anti-inflammatory response activator stm2585 gene were down-regulated in LSMMG. However, some of the delta-hfq expressed genes were oppositely regulated in LSMMG with respect to the WT, such as the D-galactonate transport dgoT gene, up-regulated in WT and down-regulated in the mutant. Other genes like SPI-1 iagB, responsible for the type III secretion system protein were down-regulated in WT and up-regulated in mutant Salmonella in LSMMG. Motility and chemotaxis genes were mostly up-regulated in the mutant strain, as shown in Figure 4b. Among SPI-2 genes, those involved in the Salmonella secretion system, such as ssaB and ssaK, and those responsible for the maintenance of Salmonella vacuolar internalization in hosts, like sifA and sifB, were down-regulated in the delta-hfq strain in response to LSMMG conditions. Bacterial infection can induce a different gene expression in a host. Indeed, Barrila et al. reported in Figure 4c the up- and down-regulation of host genes at 24 h post-infection (hpi) caused by WT and delta-hfq strains in LSMMG, compared to control culture conditions. Expression was represented as the FC (fold change) as a function of the FDR (false discovery rate). The latter represents the rate at which features considered significant are truly null and was <0.05. In the plots, values > 0.05 were significant. After Salmonella infection, mRNAs encoding for CXCL8 (IL-8) were up-regulated in host cells infected by both WT and delta-hfq strains in LSMMG conditions (Figure 4c). Other host genes involved in encoding for histone proteins (HIST1H3J, HIST1H1E, HIST1H2AG) were down-regulated in cells infected by WT in LSMMG. On the other hand, gene expression is associated with tumor and disease development, including MMP13 (matrix metallopeptidase 13), SERPINB4 (serine protease inhibitor serpin family B member 4), SOCS3 (suppressor of cytokine signaling 3) and SLC26A4 (Solute Carrier Family 26 Member 4), which were up-regulated after the infection with either the WT Salmonella or mutant strain, as shown in panel C. Finally, keratinocyte-fibroblast co-cultures are another promising 3D model for studying skin infections caused by various pathogens, including *Staphylococcus aureus* MRSA bacteria [133]. Panel D of Figure 4, proposed by Barua et al. [133], provides a better understanding of the interactions between MRSA strains and human skin after 48 h of infection. Each line, composed of i, ii, iii and iv, represents a strain of MRSA (ST8, ST30, ST59, ST22, ST45, ST239). The authors marked (i) HaCaT keratinocytes nuclei present in the strata basale and spinosum and fibroblasts localized in collagen gel. They moreover observed the dissemination of various MRSA strains (ii) through the layers of 3D skin models thanks to the interaction between bacterial FnBPs (fibronectin-binding proteins) and the epithelial cell HS60 protein (heat-shock protein 60). The staphylococcal colonization of human tissue is due to MSCRAMMs (microbial surface components recognizing adhesive matrix molecules) surface proteins, including FnBPs [134,135], that interact with human extracellular matrix proteins, causing the infections.

Barua et al. [133] evaluated not only the staphylococcal internalization from epithelial cells but also the eukaryotic cell apoptosis (iii) induced by the different MRSA strains. Panel D (iv) shows a merger of the various kinds of staining used in the study. The present model used in this study is able to more accurately mimic physiological responses and provides reliable information for observing differences in the stimulation of cell death up to 48 h, information that could not be properly retrieved from a 2D model.

New culture methods are constantly being developed, while old methods are being adapted to meet the challenges of today. A deeper understanding of the spatial and temporal interactions between microbes, hosts and their environment may aid in the development of in vitro, ex vivo and in vivo disease models and may also lead to new therapeutic approaches in different research fields.

## 6. Discussion and Concluding Remarks

As is documented in this review, there is no doubt that 3D cultures can be a formidable tool for research aimed not only at better elucidating cellular and molecular mechanisms but also at identifying therapeutic targets and testing potential therapeutic strategies. A wide variety of 3D cultures have been used, ranging from the simplest and least expensive systems, such as spheroids, to those that allow for the spatial control of cells and the modulation of certain characteristics of the cellular microenvironment (microfluidic and bioprinting techniques-based). In general, it can be argued that the 3D cell cultures mentioned in this review provided new experimental evidence on cellular and molecular mechanisms, such as those related to proliferation, differentiation, migration, gene expression signatures, microRNA profiling and growth factor signaling in support of or in combination with 2D cultures.

It is worth emphasizing that developing accurate 3D models may lead to the evaluation of a certain association between the different research areas considered here. For example, 3D cell cultures could be established with cellular and matrix components to mimic metastatic sites of breast cancer, such the liver, bone, lungs and brains [75]. Some studies hypothesized the association between endometriosis and breast cancer [136]: 3D models could help us understand the signaling interaction between cancer cells and those involved in endometriosis and whether there are possible pathogenetic pathways linking the two diseases. It is estimated that infections may contribute to up to 20% of all human tumors [137,138]): the utilization of 3D models may prove to be a valuable approach in studying the correlation between bacterial infections and cancer, as well as identifying the adaptation strategies employed by bacteria to evade antibiotic/antimicrobial therapy. In addition, the CF-Mu3Gel hydrogel, which contains mucin, has recently been developed by our research group [139]. This hydrogel could serve as a valuable 3D model for evaluating the effectiveness of new antimicrobial drugs. 

Another potential benefit of using 3D models is in the field of nanomedicine, which involves the application of nanotechnology in medicine. The similarity between spheroids or 3D models and diseased tissue can allow for screening and exploring the biological effects of different nanotherapeutics for different uses, such as chemotherapeutics, chemo-immunotherapeutics, radiotherapeutics, photothermal therapies, photodynamic therapies and gene delivery [4].

Despite the enormous potential, 3D cell cultures face many challenges, including the difficulty of replicating all the chemical and biophysical characteristics of the tissue microenvironment, the frequent use of the cell lines, the poor standardization of protocols and the higher costs compared to those of 2D systems [126]. Furthermore, the reproducibility of the results may be reduced by technical drawbacks along the quantification methods based on biochemical analysis or microscope imaging due to the complex nature of 3D cell cultures themselves. (e.g., the collection of cells or secreted factors for biochemical tests may be more difficult in some ECM-based 3D cell cultures) [140]. We believe that the publication of detailed protocols will help the research community standardize the way 3D cell cultures are set up and the way cellular and molecular effects are analyzed. Similarly, integrated analysis of the transcriptome and proteome could help determine the most appropriate model in terms of clinical translation, cost and efficiency. Beyond these considerations, in our view, the choice of the type of 3D platform/technology will undoubtedly need to be evaluated based on the scientific questions to be explored.

## Figures and Tables

**Table 1:** Summary of some recent findings on BC using 3D cell cultures.

Type of Platform	Technical Features	Cells	End-Point	Ref.
Spheroid	Agarose-based	MDA-MB-231	The cell invasion capacity increased compared to that of the 2D model; the E-cadherin expression was down-regulated while N-cadherin was up-regulated	[54]
Spheroid	Matrigel	MDA-MB-231; MCF-7; CAFs; NFs	miR-500a-5p was highly expressed in breast cancer cell lines. CAFs-derived exosomes promoted breast cancer progression and metastasis via miR-500a-5p by binding USP28	[55]
Spheroid	Agarose molds, which were cast in MicroTissues^®^3D Petri Dishes^®^	ASCs; adipocytes; MDA-MB-231; MCF-7	The model showed a direct interaction between breast cancer cells, adipocytes and ASCs. Gene expression revealed a remarkable up-regulation of CCL5 and its receptor CCR1. The CCL5/CCR1 axis promoted tumor progression when the cells were in close contact in 3D TME	[56]
Spheroid	3D collagen gels with increasing stiffness	MCF-7; HCC1954	The breast cancer spheroids model evidenced how ECM stiffness influenced cell invasion capacity. In the lower stiffness, ERK activity was increased and operated upstream of the YAP signaling, determining ECM remodeling through MMPs	[57]
Spheroid	Agarose	MDA-MB-231; HFFF2; BT474	Exosomes secreted by ADMSCs were able to deliver anti-cancer drugs, using a low concentration of Cis and PTX. Exosomes loaded with drugs increased the chemotherapy response by reducing cell viability and activating the apoptosis pathway	[58]
Organoid	Matrigel	MDA-MB-231; T-47D; MCF7	miR-93 was able to affect cell viability. The constitutive up-regulation of miR-93 suppressed invasion and the metastasis process reducing WASF3 expression	[59]
Organoid	Resuspension with matrix glue in 48-well plates	MCF-7	The tamoxifen anticancer drug inhibited the growth of MCF-7 organoids, inducing ferropstosis. Erastin, a ferrosptosis activator, enhanced the sensitivity of TAMR cells to the anticancer drug	[60]
Bioprinted Hydrogel	Small Plug Cell Model with PEG bionk formulation	MDA-MB-231; MCF-7; NHDF	DOX treatment determined cell death by entering in the 3D hydrogel and altering ERK1-ERK2 phosphorylation. Pharmacological treatment increased ATP production. GSKβ3 phosphorylation was decreased due to DOX-induced cell stress.	[61]
Hydrogel	3D silk scaffold encapsulated in a GelMA hydrogel-based hybrid system	h-ADMSCs; HUVEC; MDA-MB-231	TC-TBNC recapitulated, in a realistic way, TME. DOX and Cis treatments determined cell death through an apoptosis process: Bcl-2 was down-regulated while Bax was up-regulated. The model showed an increase in ABCC1 expression in the Cis-treated hydrogel compared to the DOX -treatment. Cis was more potent in causing a cytotoxicity effect.	[62]
Scaffold-based	Freeze-dried Silk Fibroin-Scaffold	HMF; MDA-MB-231; MCF-7	3D scaffolds were cultured in two different manners with and without fibroblasts. Cell growth was monitored, and it was higher in the co-culture model. Gene expression, related to TME, revealed that Col-I, FN, MMP1, MMP2 and MMP3 expression was higher in the heterotypic tumor culture compared to that in the homotypic one	[63]
Scaffold-based	Biomimetic collagen scaffolds	MDA-MB-231; MCF-7	MCF-7 DOX-resistant cells were characterized by an overexpression of TP53I3 and TAP1 correlated with multidrug resistance phenomena. Cells showed an enhanced expression of the GADD45 family, correlated with a reduced DNA damage response MDA-MB-231 cells reduced drug accumulation by down-regulating the endocytic pathway and activating the lysosomal pathway	[64]
Acini	GFR Matrigel^®^	MCF-10A.B2	The JNK signaling pathway was involved in HER 2^+^ breast cancer tumor progression: its deficiency promoted an acceleration of cell proliferation	[65]
Hybrid hydrogel system	Tumor spheroids surrounded by a CAF-embedded collagen -hydrogel	MDA-MB-231; MCF-7; CAFs	3D complex models were characterized by different stiffnesses. The higher expression of matrix genes RNA sequencing revealed that cells in a soft environment up-regulated YAP1, while cells in a stiffer matrix up-regulated proangiogenic proteins (FN1 and MMP9). BC cells shift from glycolysis to OXPHOS and FA metabolism, responding to a stiff microenvironment	[66]
Co-culture microfluidic tumor model	Hydrogel-based matrices injected into tumor microfluidic chips	SUM-159; MDA-MB-231; MCF-7; CAFs	The 3D co-culture model revealed that CAFs enhanced breast cancer proliferation. The model showed the central role of GPNMB in tumor progression: the knockdown of this protein was able to reduce the effects of CAFs on cancer invasion	[67]

**Abbreviations.** GFR: Growth factor reduced; JNK: c-Jun N-terminal kinases; ECM: Extracellular matrix; YAP: Yes-associated protein; CAFs: Cancer-associated fibroblast; USP28: Ubiquitin-specific peptidase 28; COL1A1: Collagen type I alpha 1 chain; COL3A: Collagen type I alpha 3 chain; Col-I: Collagen type I; FN: Fibronectin; MMP1: Metalloproteinase-1; MMP2: Metalloproeinase-2; MMP3: Metalloproeinase-3; HMF: Human mammary fibroblasts; GPNMB: Glycoprotein non-metastatic B; NFs: Normal fibroblasts; WASF3: Wiskott–Aldrich syndrome protein family member; ASCs: Adipose-derived stromal cells; CCL5: C-C motif chemokine ligand 5; CCR1: C-C chemokine receptor type 1; TME: Tumor microenvironment; DOX: Doxorubicin; NHDF: Neonatal human dermal fibroblasts; TP53I3: Tumor protein p53-inducible protein 3; TAP1: Transporter associated with antigen processing 1; GADD45: Growth arrest and DNA damage; h-ADMSC: Human adipose-derived mesenchymal stem cells; Cis: Cisplatin; PTX: Paclitaxel; ERK1: Extracellular signal-regulated kinases 1; ERK2: Extracellular signal-regulated kinases 2; PEG: Polyethylene glycol; GSKβ3: Glycogen synthase kinase 3 beta; SF: Silk fibroin; GelMA: Gelatin Methacryloyl; TAMR: tamoxifen-resistant MCF-7; TC-TBNC: Triculture triple-negative breast cancer, Bax: Bcl-2 Associated X protein; OXPHOS: oxidative phosphorylation; FA: fatty acid metabolism.

**Table 2:** Summary of key 3D cell culture models for human endometriosis.

Type of Platform	Technical Features	Cells	End-Point	Ref.
Spheroid	polyHEMA coated multiwell	EEC16, EEC16	Up-regulation of the gene involved in the immune response and hormonal signaling	[86]
Spheroid	hanging-drop method	St-T1b	Up-regulation of the gene involved in invasion and EMT	[87]
Spheroid	Kenzan method	12Z	Up-regulation of the gene involved in the inflammatory response and hormonal signaling	[88]
Spheroid	micro-molded agarose well plates	12Z, iEc-ESCs, iHUFs	Up-regulation of the gene involved in the immune, inflammation and invasion process	[89]
Tissue slices	3D air–liquid interface cultures-collagen type I	Primary endometrial cells	Up-regulation of the gene involved in cell proliferation and hormonal signaling	[90]
Organ on a chip	microfluidic 3D tri-culture model	HUVECs; Ishikawa; ESFs: CRL-4003 cells	Up-regulation of the gene involved in angiogenesis	[91]
Organoid	liquefied growth factor reduced Matrigel	Primary endometrial cells	Epigenetic modification for the HOX genes and their cofactors	[92]

**Abbreviations.** EEC16: ovarian endometriosis epithelial cell line; St-T1b: immortalized endometrial stromal cells; EMT: epithelial–mesenchymal transition; 12Z: endometriotic epithelial cells; iEc-ESCs: immortalized endometriotic stromal cells; iHUFs: immortalized uterine stromal; HUVECs: human umbilical vein endothelial cells; Ishikawa: endometrial epithelial cells; ESFs: CRL-4003 cells: endometrial stromal fibroblasts; HOX: Human Homebox.

**Table 3:** Summary of recently developed 3D hepatic cultures based on cell lines and primary cells.

Type of Platform	Technical Features	Cells	End-Point	Ref.
Spheroid	Matrigel-embedded spheroids	HepG2	Enhanced secretion of albumin and urea. Up-regulation of genes involved in drug metabolism (CYP450 enzymes)	[102]
Spheroid	Fabricated with a commercial system based on the hanging drop principle (GravityPLUS, Insphero)	Monoculteres of either HepaRG or HepG2	Enhanced secretion of albumin and urea. Up-regulation of genes related to drug metabolism (CYP1A2, CYP2B6, CYP3A4), gluconeogenesis (G6Pase, PEPCK2), glycolysis (L-PK), energetic lipid synthesis (SREBF1, SCD1, DGAT2), bile acid metabolism (CYP7A1, CYP8B1, ABCB11) and lipoprotein metabolism (ApoE, ApoA-1)	[105]
Bioprinted cell-laden scaffold	3D structure printed with a GelMa-based bioink	HepaRG	Enhanced albumin and urea secretion. Up-regulation of genes related to phenotypical stability. Up-regulation of phase-1 drug metabolizing enzymes (CYP1A2, CYP2B6, CYP3A4)	[104]
Bioprinted cell-laden scaffold	Pinewood structure bioprinted with a custom alginate-based bioink	HepG2	Up-regulation of genes involved in hepatic functionality (ALB), lipid metabolism (ApoA4, ApoC3) and cell proliferation (VTN)	[101]
Bioprinted cell-laden scaffold	Hexagonal structures bioprinted with an ECM-based custom bioink	HepG2	Enhanced secretion of albumin and urea	[100]
Hollow fiber bioreactor	Commercial device (C2011, FiberCell Systems)	HUH7.5	Enhanced metabolic activity	[106]
Bioprinted cell-laden scaffold	Core-shell structures printed with a custom ink based on methacrylated alginate and Matrigel. The coaxial structure enabled the segregated coculture of different cell types	HepG2 NIH 3T3	The coculture with fibroblasts enhanced albumin and urea secretion and proliferation and promoted the aggregation of HepG2	[108]
Spheroid	Self-assembly in ultra-low-adhesion U-bottom plates	HepaRG 3T3-J2	The coculture enhanced albumin and urea secretion and incremented the metabolic activity of HepaRG	[110]
Mixed	This study exploited self-assembled spheroids in U-bottom, agarose-coated wells and cells seeded in a 3D hollow fiber bioreactor	iPSCs-derived hepatocytes.	Enhanced differentiation performances in both 3D cultures (down-regulation of pluripotency markers, up-regulation of hepatic markers ALB, CYP34A and HNF4A, metabolic shift toward oxidative phosphorylation). Bioreactor-based cultures outperformed spheroids	[116]
Spheroid	The formation of spheroids was guided and achieved in a custom microfluidic device	Primary hepatocytes derived from humanized chimeric mice and murine embryonic fibroblasts (H9 SOX17-mCHERRY)	Long-term (33 days) 3D cultures of functional and polarized primary hepatocytes with enhanced albumin, urea and bile acids synthesis compared to that of 2D cultures. Promoted the differentiation of murine embryonic fibroblasts when cocultured with primary hepatocytes in the microfluidic device	[115]
Spheroid	Cell aggregation into spheroids was achieved by using custom-made agarose micro-wells arrays	iPSCs derived from iPS(foreskin)-3 fibroblasts	Successful differentiation into functional hepatocytes (albumin and urea synthesis, expression of active CYP450 enzymes)	[117]

**Abbreviations.** CYP450: cytochrome P450; CYP1A2: cytochrome P450 1A2; CYP3A4: cytochrome P450 3A4; CYP2B6: cytochrome P450 2B6; CYP7A1: cytochrome P450 7A1; CYP8B1: cytochrome P450 8B1; G6Pase: glucose 6-phosphatase; PEPCK2: phosphoenolpyruvate carboxykinase 2; L-PK: pyruvate kinase; SREBF1: sterol regulatory element-binding transcription factor 1; SCD1: stearoyl-CoA desaturase-1; DGAT2: diacylglycerol O-acyltransferase 2; ABCB11: ATP Binding Cassette 11; ApoE: apolipoprotein E; ApoA-1: apolipoprotein A1; ALB: albumin; ApoA4: apolipoprotein A4; ApoC3: apolipoprotein C3; VTN: vitronectin; HNF4A: hepatocyte nuclear factor 4 alpha.

**Table 4:** Examples of 3D in vitro models for the study of host–microbe interactions.

Type of Platform	Technical Features	Pathogens	End-Point	Ref.
3D gut epithelium	Microchannel-embedded hydrogel	co-cultures of *Salmonella enterica* and *Lactobacillus reuteri*	Overexpression of NF-κB and TNF signaling pathways; Rap1 signaling; homologous recombination in the Caco-2 cells co-cultured with pathogens	[127]
3D human endocervical model	RWV bioreactor technology	*L. crispatus*, *A. vaginae*, *G. vaginalis*, *P. bivia*, *S. amnii*	Exhibition of the pro-inflammatory potential through the induction of specific cytokines (e.g., IL-6, IL-8, INF-ϒ-induced protein-10, monocyte chemotactic protein 10), iNOS and oxidative stress-associated compounds	[128]
Model of the small intestine	Transwell	*E. faecalis*, *E. coli*, *S. salivarius*, *S. mitis*, *L. plantarum*, *V. parvula*, *V. atypica* and *P. intermedia*	Secretion of IL-6, IL-8, TNF-α, and CXCL16; Protein expression (Actin); Gene expression (TLR-2 and -4 and DUOX2)	[129]
Gut organoid	Transwell	*Listeria monocytogenes*	Bacterial translocation study (increase in the pro-inflammatory response: IL-8, TNF-α)	[130]
Enteroids	Human intestinal enteroids (HIE)	*Shigella flexneri*	Increased HIE proinflammatory signals and the amino acid transporter SLC7A5	[131]
Hollow fiber bioreactor	3D model of human colonic epithelial cells (HT-29) cultured on bioreactors (with/without macrophages) in microgravity	*Salmonella enterica* (serovar Typhimurium)	Increased bacterial virulence in an spaceflight analogue culture (expression of adherence, invasion, motility and chemotaxis genes); Increased expression of host inflammatory genes (IL-8)	[132]
3D organotypic skin model	HaCaT keratinocyte-fibroblast co-cultured on transwell	*Staphylococcus aureus* MRSA strains	Assessment of MRSA pathogenicity and HaCaT/fibroblasts DNA damage detection	[133]

**Abbreviations.** NF-κB: Nuclear Factor kappaB; TNF: Tumor Necrosis Factor; Rap1: Ras-associated protein 1; Caco-2: human colorectal adenocarcinoma cells; IL: interleukin; INF-ϒ: interferon gamma; iNOS: inducible nitric oxide synthases; CXCL16: chemokine (C-X-C motif) ligand 16; TLR: Toll-like Receptor; DUOX2: dual oxidase 2; HIE: human intestinal enteroids; SLC7A5: solute carrier family 7 member 5; HT29: human colon adenocarcinoma cell line; HaCaT: human keratinocyte cell line; MRSA: methicillin-resistant *Staphylococcus aureus*.

## Data Availability

Not applicable.
